# Effects of Ferroptosis on Cardiovascular Diseases

**DOI:** 10.1155/2023/6653202

**Published:** 2023-05-04

**Authors:** Jiayi Xu, Jinkui Pi, Yanjing Zhang, Jinhan Zhou, Shuxia Zhang, Sisi Wu

**Affiliations:** Core Facilities, West China Hospital, Sichuan University, Chengdu, Sichuan 610041, China

## Abstract

Ferroptosis is a novel form of programmed cell death characterized by the accumulation of iron-dependent lipid peroxides, which causes membrane injury. Under the catalysis of iron ions, cells deficient in glutathione peroxidase (GPX4) cannot preserve the balance in lipid oxidative metabolism, and the buildup of reactive oxygen species on the membrane lipids leads to cell death. An increasing body of evidence suggests that ferroptosis plays a significant role in the development and occurrence of cardiovascular diseases. In this paper, we mainly elaborated on the molecular mechanisms regulating ferroptosis and its impact on cardiovascular disease to lay the groundwork for future studies on the prophylaxis and treatment of this patient population.

## 1. Introduction

Dixon first reported that ferroptosis is a kind of iron-dependent programmed cell death in 2012 that differs from apoptosis, necrosis, and autophagy [1].

Current evidence suggests that the overoxidation of polyunsaturated fatty acids (PUFAs) and the limited ability of glutathione peroxidase 4 (GPX4) to neutralize oxalate phospholipids are important mechanisms of ferroptosis. In the presence of iron ions, the Fenton reaction intensifies reactive oxygen species (ROS) formation, leading to lipid peroxidation, iron accumulation, membrane oxidation damage, and finally, programmed cell death. Three key factors of ferroptosis have been documented: PUFAs, ferrous ions (Fe^2+^), and GPX4 malfunction [1, 2].

Compared with other types of programmed death, ferroptosis has the following characteristics.

1. Cell morphology changes occur; ferroptosis leads to abnormal mitochondria, an increase in the membrane density of mitochondria, a decrease or loss of cristae, rupture of the outer membrane, and damage to other mitochondrial bodies and functions. A loss of membrane integrity, with bubbles or cracks, may be observed. The cell morphology is normal, with no internal chromatin agglutination
2. Changes in cell composition are observed, indicating that ferroptosis results in metabolic abnormalities such as the reduction of GSH synthetic raw materials, diminished or inactivated GPX4 expression, damage to the cysteine uptake system, increased iron and ROS content, and enhanced lipid peroxidation and oxidative stress [3–5].

## 2. The Molecular Mechanism of Ferroptosis

### 2.1. Amino Acid Metabolism Imbalance

It is well established that ferroptosis is controlled by amino acid metabolism. Glutamine is the most widely used amino acid in vivo and exhibits the highest concentrations in human tissue and plasma. In addition to being involved in tricarboxylic acid (TCA) cycle intermediates, glutamine is vital for lipid biosynthesis. Glutamine can be hydrolyzed to glutamic acid, one of the most important substrates for glutathione synthesis (GSH) [6]. GSH is composed of glutamic acid, cysteine, and glycine, and mammalian cells rely on it as the most important nonenzymatic antioxidant. GSH exists in the cytoplasm in concentrations between 85% and 90%, providing electrons for peroxide reduction under the catalysis of glutathione peroxidase (GPx). GSH can produce an oxidized GSH dimer (GSSG) when it directly reacts with ROS [7]. GSH and GSSG constitute the main antioxidant system of cells, and GSH participates in cell proliferation and immune response regulation through the MAPK and ERK pathways. Activation of the GSH/MAPK pathway induces ferroptosis in human islet cells, and inhibition of the MAPK pathway can significantly inhibit inflammation and oxidative stress reactions caused by ferroptosis [8, 9]. The decrease in GSH leads to increased content of lipid ROS in cells, which contributes to damage to various proteins or cell membranes and the subsequent death of iron-dependent cells [10]. Cysteine plays a key role in GSH synthesis and is directly related to its intake. Cysteine deficiency can lead to glutathione depletion, resulting in the inactivation of GPX4. After the inactivation of GPX4, potentially toxic lipid hydroperoxide cannot be converted into nontoxic lipid alcohol, which can directly affect the degradation of intracellular lipid peroxide products [11, 12]. As the extracellular cystine levels are higher than intracellular levels, cystine is transported into cells, and intracellular glutamic acid has a higher concentration relative to extracellular glutamic acid, resulting in glutamic acid output from cells [13]. When glutamine decomposition is inhibited or deficient, cystine starvation induces ROS accumulation, lipid peroxidation, and ferroptosis.

It has been established that intracellular cysteine levels are regulated by a cystine/glutamic acid reverse transport system, System Xc-, which consists of solute transport family 7A11 (SLC7A11) and SLC3A2. Glutamate and cystine are transported from the intracellular space to the extracellular space by System Xc- and affect GSH synthesis through intracellular cystine. Inhibition of System Xc- can directly expropriate the cells of cysteine and repress the synthesis of GSH. Therefore, System Xc- is the most important target process of compound-induced ferroptosis [14, 15].

A study based on a disulfide-linked light-chain xCT gene knockout mouse model found that cystine/cysteine in plasma is often in an oxidative state, which indicates that system Xc- also plays an important role in maintaining the dynamic balance of cell oxidation and reduction by mediating the oxidation-reduction reaction of cystine/cysteine [16].

The mitochondrial electron transport chain (ETC) expedites ferroptosis induced by cysteine deletion. When the level of cysteine is reduced and the catabolism of glutamine is blocked, mitochondrial metabolism can promote glutathione consumption in cells. As a result, the mitochondrial membrane potential becomes hyperpolarized, lipid peroxide gradually accumulates, and ferroptosis occurs [17]. However, the deactivation of fumaric acid hydrate (a component of the TCA cycle) and inhibition of the mitochondrial TCA cycle, or ETC, can transform the above reaction and enhance the resistance to ferroptosis induced by cysteine deprivation. NRF2 can directly or indirectly regulate the expression of the GPX4 protein and mediate the expression of SLC7A11 and GSS to promote GSH synthesis. GSH is converted by GPX4 to GSSG to ameliorate intracellular lipid hydrogen peroxide levels [18]. Erastin, a ferroptosis inducer, can inhibit cystine input, affect glutathione synthesis, inactivate GPX enzymes in cells, and induce cell oxidation–reduction disorders, eventually leading to cell death [12]. Current evidence suggests that erastin-induced ferroptosis coupled with transcription factor 3 (ATF3) can inhibit SLC7A11 expression and System Xc- action by binding to the SLC7A11 promoter, mediating GSH depletion in cells, and promoting ferroptosis in cells [19]. Several cell types can acquire cysteine through other means, thereby gaining resistance to cell death induced by erastin. For instance, cysteinyl-tRNA-knockdown cells can transform cystathionine to cysteine by upregulating the sulfur pathway, inhibiting erastin, restoring the formation of lipid ROS, and reducing the sensitivity of cells to ferroptosis [20, 21].

### 2.2. Lipid Metabolism Imbalance

Lipid metabolism has a powerful effect on sensitivity to ferroptosis, and lipid peroxide accumulation is the core factor of ferroptosis. Lipid peroxides are formed by enzymatic reactions involving metabolic catalysis of PUFAs and nonenzymatic reactions based on redox lipid peroxidation [22].

PUFAs are an important component of cell and organelle membrane phospholipids, and they mediate inflammation, immunity, synaptic remodeling, cell growth, and other biological functions. The accumulation of large amounts of lipid peroxides and their toxic metabolites causes changes in the lipid microenvironment of numerous membrane receptors, membrane proteases, and ion channels on the plasma membrane, changes the physical properties of the cytomembrane, reduces the fluidity of the cell membrane, increases the permeability of the membrane, disrupts the ion gradient, slows down lateral diffusion, and destroys the stability of the phospholipid bilayer, conducive to the disintegration of the cytomembrane. In addition, lysosomal membrane lipids may be peroxidized due to ROS accumulation and iron overload, and the infiltration of lysosomal membranes may lead to an increase in oxygen free radicals, cell membrane degeneration, and GSH depletion [23, 24].

ACSL4, a member of the long-chain family of acyl-CoA synthetases, is a fatty acid metabolizing enzyme that can convert PUFAs such as arachidonic acid (AA) and adrenergic acid (AdA) into PUFA-containing phospholipids. Under oxidative or energy pressure, the upregulation of ASCL4 expression increased the PUFA content in phospholipids. ROS promote the oxidation of PUFAs in the bilayer of membrane phospholipids, mediating lipid peroxidation and inducing ferroptosis to maintain redox homeostasis. When the activity or expression of ASCL4 is inhibited, the synthesis of PUFAs decreases, and a reduction in ferroptosis sensitivity is observed in tissues or cells [25–27]. In an ischemia-reperfusion (IR) mouse model, the content of ASCL4 and ROS in lung tissue was found to be significantly increased. Lipoxstatin-1 could directly inhibit the effect of lipid hydroperoxide on the phospholipid bilayer of the cell membrane, significantly reduce lipid peroxidation damage in tissues and cells, and improve the damage to IR lung tissue and cells in mice [28]. However, the monounsaturated fatty acids (MUFAs) activated by ACSL3 can reduce the PUFA content in phospholipids by displacing PUFAs from phosphatidylethanolamine and inhibiting lipid peroxidation by reducing the available substrates of lipid peroxidation, thus improving cell sensitivity to ferroptosis [29].

Ferroptosis is negatively regulated by the NADPH/FSP1/CoQ10 pathway. The lack of NADPH regulates the low expression of GSH and thioredoxin (TRX) and promotes the high expression of lipid peroxidation. It has been reported that NRF2 could increase NADPH and inhibit lipid peroxidation and ferroptosis through the pentose phosphate pathway [30]. Kagan et al. found that iron-containing oxidants such as lipoxygenase (LOX) can promote cell ferroptosis induced by RSL-3 or erastin by driving the reaction between arachidonic acid and epinephrine phosphatidylethanolamine in the endoplasmic reticulum (ER) and thus participate in the peroxidation of PUFAs. Interestingly, the gene deletion of LOX has been reported to prevent erastin-induced ferroptosis. An increasing body of evidence suggests that inhibitors of LOX, including flavonoids and the vitamin E family, could inhibit cell ferroptosis by inhibiting LOX-catalyzed phospholipid peroxidation [11, 31, 32]. By covalently conjugating with GPX4, RSL-3, a ferroptosis inducer, inhibits the normal function of GPX4, thus reducing the volume of mitochondria and the number of mitochondrial cristae, precipitating the destruction of the mitochondrial outer membrane and the accumulation of lipid peroxide, thus leading to ferroptosis [32, 33]. Current evidence suggests that upregulating GPX4 expression can effectively reduce the injury induced by inflammatory conditions by restraining the nuclear factor-*κ*B (NF-*κ*B) pathway and the oxidation of AA, lowering the level of ROS, and inhibiting ferroptosis [34].

Current evidence suggests that a voltage-dependent anion channel called VDAC is activated by erastin by binding to it, which increases the mitochondrial membrane potential, encourages hyperpolarization, alters outer membrane permeability, accelerates ROS production in mitochondria and cells, induces the abnormal structure and function of mitochondria, and promotes cell ferroptosis. [14, 35, 36].

### 2.3. Iron Metabolism Disorder

As an essential component of physiological activities such as cell proliferation, cell oxygen transport, energy metabolism, DNA synthesis, and DNA repair, iron is of great significance to maintaining normal physiological function and metabolic homeostasis. Transferrin (TF), solute carrier family 40 member 1 (SLC40A1), and solute carrier family 11 member 2 (SLC11A2) are essential for maintaining intracellular iron concentrations. Iron is oxidized under two conditions: ferrous ions (Fe 2+) or ferric ions (Fe 3+). By binding to transferrin, extracellular Fe3+ is recognized by transferrin receptor 1 (TFR1) on the cytomembrane and transported intracellularly. The metal reductase STEAP3 reduces it to Fe2+, and the transporter DMT1 transports Fe2+ into an unstable iron pool via SLC11A2. The excess iron is released into the blood or temporarily kept in ferritin [37].

Under pathological conditions, the stable state of iron metabolism is destroyed, which can lead to the import of iron from the extracellular environment by transferrin and the transferrin receptor, the reduction of cellular ferritin, and the multiplication of transferrin, leading to increased Fe2+ content in the cytoplasm. During iron overload, many hydroxyl radicals are generated utilizing the Fenton reaction, which indirectly or directly participates in the production of ROS, inducing lipid peroxidation and the accumulation of infusibility proteins in the ER. These substances mediate a series of oxidation and peroxidation reactions to destroy DNA, proteins, lipids, and other biomolecules. A host of lipid ROS is generated when they directly interact with PUFAs in the cytomembrane, which promotes cell death [38, 39]. Iron homeostasis can be controlled by lysosomes, which promote the production of ROS [40]. It is widely thought that mitochondrial ferritin protects ferroptosis, given that overexpressed ferritin in mitochondria exhibits resistance to erastin-induced ferroptosis [41]. mitoNET and NAF1 are involved in mitochondrial iron transport. Overexpression of mitoNET inhibits ferroptosis in human hepatoma cells induced by erastin, and overexpression of NAF1 may also reduce ferroptosis induced by sulfasalazine [42, 43].

A study established a DOX-induced cardiomyopathy mouse model and found that heme could be rapidly degraded by upregulating heme oxygenase-1 (Hmox1), and iron was released into the bloodstream more swiftly, leading to myocardial dysfunction and ferroptosis [44]. NRF2 can regulate the level of free intracellular iron. Heme synthesis can be regulated by ABCB 6, and the output of iron-containing porphyrin promotes iron depletion. Iron homeostasis is also regulated by intracellular NRF2 mediated by promoting the expression of TfR1, FTH, FTL, and FPN [45, 46].

**Figure 1:**
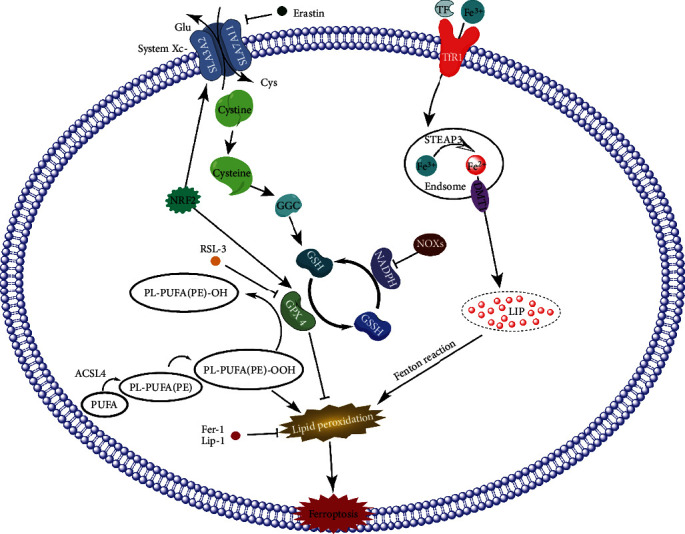
Schematic representation of the mechanism of ferroptosis. Dysfunction of PUFA, Fe2+, and GPX4 is the key factor in iron death. An increase in free iron and a decrease in GPX4 will induce iron death through lipid peroxidation. Glu: glutamate; Cys: cysteine; GGC: gamma-glutamylcysteine; GSH: glutathione; GPX4: glutathione peroxidase 4; GSSG: oxidized glutathione; Nrf2: nuclear factor-erythroid-2-related factor 2; ACSL4: acyl-CoA synthetase long chain family member 4; PUFA: polyunsaturated fatty acid; PL-PUFA(PE): phospholipids containing polyunsaturated fatty acids; PL-PUFA(PE)-OOH: phospholipid hydroperoxide containing polyunsaturated fatty acid; TF: transferrin; TfR1: transferrin receptor 1; STEAP3: six-transmembrane epithelial antigen of prostate 3; DMT1: divalent metal-ion transporter-1; Lip: labile iron pool; Fer-1: ferrostatin-1; Lip-1: liproxstatin-1; and RSL3: RAS- selective lethal 3.

The three core factors of ferroptosis, the dysfunction of GPX4, PUFA, and Fe2+, are shown in Figure 1. Dysregulated iron regulation can increase intracellular free iron; the Fenton reaction is catalyzed by sufficient free iron to form ROS, and ROS can further promote lipid ROS reactions, causing lipid peroxide aggregation and inducing ferroptosis. System Xc-, a cystine/glutamic acid transporter located in the cytomembrane, participates in the synthesis of GSH. GPX4 converts lipid peroxides to normal lipids, prevents their accumulation, and inhibits ferroptosis by using GSH as its substrate. Erastin inhibits System Xc- which can deprive cells of cysteine, leading to GSH depletion, which causes the inactivation of GPX4. RSL-3 can directly inhibit the activity of GPX4, affect the accumulation of intracellular lipid peroxides, and induce iron death. Overall, these findings suggest that ferroptosis is induced by lipid peroxide accumulation due to excessive lipid peroxidation and the inability of GPX4 to scavenge peroxides [47].

## 3. Effects of Ferroptosis on Cardiovascular Disease

Cardiovascular diseases represent a common cause of mortality, including hypertension, atherosclerosis, acute myocardial infarction (AMI), cardiomyopathy, valve disease, and heart failure [48, 49]. Recent research has revealed an association between ferroptosis and cardiovascular disease. Cardiomyopathy, myocardial infarction, ischemia/reperfusion injury, and heart failure are linked to ferroptosis [50]. Cardiovascular disease may be treated effectively by inhibiting ferroptosis and thus preventing the death of cardiac cells.

### 3.1. Myocardial Ischemia-Reperfusion Injury (MIRI)

It is widely acknowledged that during ischemic heart disease, coronary atherosclerotic plaques occlude the coronary arteries, causing thrombosis complications and biochemical and metabolic changes, ultimately leading to myocardial cell death. Ischemia-reperfusion injury (IRI), which may occur during treatment, can significantly hinder the treatment efficacy. There is an increasing consensus that myocardial reperfusion injury is characterized by the accumulation of iron and the production and increased ROS and reactive nitrogen species (RNS) levels. During ischemia/reperfusion, ROS and RNS are increased, leading to enhanced oxidative and nitrosation stress, causing damage to the body. In addition, the dysregulated iron homeostasis can cause an increase in free iron in cardiac myocytes and mediate the increase in hydroxyl free radicals through the Fenton reaction [47, 51]. Various stress products caused by myocardial ischemia and reperfusion damage cells and organisms by attacking biomolecules such as lipids, DNA, and protein and activating various cellular death pathways (such as apoptosis, necrosis, apoptosis, and ferroptosis).

It has been established that two kinds of serum factors, transferrin and glutamine, can stimulate cell necrosis during ischemia. This type of serum-dependent necrosis has been reported as ferroptosis. In isolated mouse hearts subjected to ischemia-reperfusion, cellular death caused by cystine starvation and the subsequent imbalance of cell redox homeostasis can be inhibited by a ferroptosis inhibitor (Fer-1), iron chelating agents (DFO and CPX), and the glutamine decomposition inhibitor compound 968 to improve heart ischemia/reperfusion injury [52]. There is a rich literature available substantiating that myocardial ischemia and reperfusion increase the production of oxidized phosphatidylcholine (OxPC). OxPC can produce bioactive phospholipid intermediates through oxidation reactions, destroy mitochondrial bioenergetics and calcium transients, and cause extensive cell death through ferroptosis. Fer-1 can inhibit the ferroptosis of myocardial cells during reperfusion caused by OxPC [53]. Ubiquitin-specific protease 7 in myocardial tissue is significantly increased after ischemia-reperfusion, which mediates the increase in the expression of the human tumor suppressor gene p53 protein and TfR1. TfR1 can regulate ferritin accumulation in the damaged myocardium and induce ferroptosis and myocardial injury. By deubiquitinating p53, the expression of TfR1 can be significantly reduced to inhibit myocardial cell ferroptosis [54, 55].

NRF2 is a crucial regulator of the cellular antioxidant response that regulates the antioxidant response of various cells mainly by protecting cells from lipid peroxidation stress and iron metabolism homeostasis, inhibiting ferroptosis, and relieving myocardial I/R injury through ferroportin 1, an iron-releasing related protein [5, 56]. Current evidence suggests that DOX, an anticancer drug, can upgrade heme oxygenase-1 (HMOX-1) through NRF2, degrade heme and release more Fe2+, and downregulate the expression of GPX4 through System Xc-, which mediates lipid peroxides amassable within mitochondria and cells, regulates the expression of total iron and Fe2+ in myocardial tissues, inflammatory reaction and lipid peroxidation accumulation, induces mitochondrial and cellular ferroptosis, and applies naringenin NAR to regulate the role of iron-ATP in Fenton's reaction by Nrf2/System Xc-/GPX4 pathway, reducing inflammation and lipid peroxidation, activating ferroptosis-related proteins, and improving myocardial I/R injury caused by ferroptosis in rats. Interestingly, using iron chelators to overexpress GPX4 or target Fe2+ or ferroptosis inhibitors can significantly improve doxorubicin-induced cardiac damage [44, 57, 58].

### 3.2. Myocardial Infarction

Acute myocardial infarction (AMI) refers to local myocardial damage caused by severe myocardial ischemia and hypoxia caused by coronary artery lesions [59]. Myocardial infarction is closely associated with hypoxia-related genes such as SELP, CXCL2, MyD88, and S100a8 and genes related to Atf3, PTGS2, Cxcl1, and Socs3 ferroptosis. Moreover, several hypoxia-related genes, including CXCL2 and Myd88, and ferroptosis-related genes, such as ATF3 and Ptgs2, have been linked to MI development and occurrence [60]. It has been shown that CXCL2 can mediate the inflammatory reaction and regulate AMI [61]. MyD88 mediates the TLR4/NF-*κ*B inflammatory pathway to prevent isoproterenol- (ISO-) induced AMI, and inhibiting MyD88 expression prevents pathological left ventricular remodeling and alleviates myocardial infarction injury [62, 63]. This process of ferroptosis regulated by GPX4 is accompanied by PTGS2, a sign of lipid peroxidation [12]. Besides, ATF3 stimulates ferroptosis by inhibiting SLC7A11 in a p53-independent manner [19].

Quantitative protein omics analysis showed that GSH, fatty acid, redox, and epithelial-mesenchymal transition (EMT) pathways were downregulated during early myocardial infarction. The ROS pathway, glycolysis, and fatty acid metabolism were downregulated in the intermediate phases, and related changes such as translation inhibition, RNA splicing, and mRNA processing were observed in the late stages. During the early and intermediate stages of myocardial infarction, inflammation and immune pathways are activated due to cysteine deprivation, and GSH and GPX4 levels are significantly decreased. Myocytes from neonatal rats become susceptible to ferroptosis during GPX4 inhibition [64]. EMT and NRF2 have been associated with myocardial infarction. Current evidence suggests that overexpression of EMT-labeled ZEB1 or E-cadherin (CDH1) silencing increases the susceptibility of cells to ferroptosis. ZEB1 regulates lipid metabolism, affects EMT-related plasma membrane remodeling, and directly inhibits GPX4 biosynthesis and lipid peroxidation to affect ferroptosis [65, 66]. During myocardial infarction, the EMT pathway is activated, and EMT is positively correlated with ferroptosis in cardiac cells [67]. During the early and intermediate phases of myocardial infarction, Hmox1 activity is reportedly enhanced and mediated by the Nrf2/Hmox1 axis, which promotes iron overload and leads to ferroptosis in myocardial cells [5]. Human umbilical cord blood exosomes may inhibit the expression of DMT1 through MIRI-23A-3P, thus assisting in the prevention of ferroptosis and alleviating the effects of acute myocardial infarction on the heart [68]. Ferroptosis inhibitors can reduce the range of ischemic infarction by reducing the expression of VDAC protein 1 in the mitochondrial outer membrane, increasing the content of the antioxidant GPX4 in the mitochondria, and reducing the accumulation of ROS in the mitochondria.

### 3.3. Atherosclerosis

Atherosclerosis (AS) is the pathological basis for various vascular diseases characterized by partial arterial wall lipid deposition. Atherosclerotic plaques narrow the arteries, mediated by the proliferation of smooth muscle cells and fibrous matrix [69–71]. Research has shown that AS is associated with lipid peroxidation, inflammation, and iron deposition [72, 73]. Ferroptosis regulates atherosclerosis and plays a crucial role in the progression of early AS to late AS, with endothelial cell ferroptosis accelerating AS [74].

The use of ferroptosis inhibitors has been reported to upregulate the expression of SLC7A11 and GPX4, significantly reduce lipid peroxidation and iron content in mouse aortic endothelial cells, improve cell lipid peroxidation and endothelial dysfunction, and alleviate AS damage [75]. During the late stage of atherosclerosis, the levels of the iron ferroptosis-related proteins PTGS2 and ACSL4 are increased, while GPX4 expression is decreased. Positive correlations were observed between AS severity and the expression levels of PTGS2 and ACSL4, and negative correlations between AS severity and GPX4 expression. These findings suggest that PTGS2 may be a core gene in atherosclerosis [72]. An increase in lipoxygenase (LOX) expression has been shown to promote atherosclerosis, while a decrease in 12/15-LOX prevents the deposition of oxidized low-density lipoprotein under the endothelium and delays atherosclerosis development [76]. Ferroptosis and atherosclerosis are regulated by GPX4 [74]. This protein downregulates LOX and cyclooxygenase (COX) in addition to inhibiting AA oxidation and activation of the NF-*κ*B pathway to reduce proinflammatory factor levels, reduce ROS production, and inhibit the release of inflammatory mediators and inflammatory responses related to ASAS [77, 78]. Activated NRF2 can improve oxidative stress in the body. In the presence of NRF2, NADPH is produced more readily, relieving the decrease in the expression of GSH and TRX caused by NADPH deficiency, regulating the synthesis of GPX4, and inhibiting lipid ROS accumulation and cell ferroptosis [30]. High uric acid levels can prevent the NRF2/SLC7A11/GPX4 axis and increase iron overload and lipid peroxidation. Fer-1 can upregulate the level of protective factors related to ferroptosis, improve iron and lipid peroxidation accumulation, and downregulate the expression of proinflammatory factors to reduce the death of endothelial cells and improve endothelial cell activity [75, 79].

### 3.4. Cardiac Hypertrophy-Heart Failure

Heart contraction defects or impaired filling will seriously damage the heart's capacity to pump blood to other body parts, leading to heart failure. Therefore, cardiomyopathy is a major cause of heart failure. The mortality rate of cardiomyocytes in heart failure is significantly higher than nonfailing cardiomyocytes, indicating that cardiomyocyte death is a fatal pathogenic component of the pathophysiological process of heart failure [50].

Free iron in the myocardium is a critical risk factor for heart failure following acute myocardial infarction. Iron homeostasis is crucial for the myocardium and heart to continue functioning properly. FPN is an iron transporter expressed in myocardial cells and is at the core of iron homeostasis. It has been established that iron overload can induce hypertrophic cardiomyopathy and heart failure [80]. Taken together, FPN and TfR1 control how much iron is absorbed and how ferritin is stored. The destruction of the HAMP/FPN pathway of ferritin mediates the iron overload in the heart, resulting in cardiac myofibrillar disorder and an increase in mitochondria in myocardial cells. These mice developed dilated cardiomyopathy with left ventricular dysfunction. Intravenous iron replacement therapy can increase the intake and utilization of iron by cardiac myocytes and increase the regulation of circulating ferritin on the FPN of cardiac myocytes to correct cardiac metabolic and contractile dysfunction [81, 82]. After the removal of ferritin FTH, the expression of SLC7A11 and GSH is inhibited, stimulating an increase in lipid peroxidation, conducive to myocardial injury and heart failure. Both the overexpression of SLC7A11 in cardiomyocytes and the application of the ferroptosis regulator Fer-1 may prevent the death of heart cells and serious cardiac damage and hypertrophic cardiomyopathy induced by a high-iron diet [83]. In addition, the expression of TLR4 and NADPH oxidase 4 (NOX4) was significantly decreased in the heart tissue of rats after heart failure, and silencing TLR4 and NOX4 reduced oxidative stress and ferroptosis in heart cells and inhibited ferroptosis and heart failure mediated by the TLR4/NOX4 pathway [84]. QSG, a traditional Chinese medicine, has been reported to inhibit the release of spleen mononuclear cells, mediate the TLR4/MyD88/NF-B network, and protect the myocardium in mice with heart failure [85].

Lipid peroxidation, an elevation in the unstable iron pool, and cardiomyocyte ferroptosis have been documented in a rat model of heart failure. Using puerarin can inhibit increased iron overload and lipid peroxidation, reduce cell ferroptosis, and improve myocardial cell loss in heart failure caused by pressure overload [86]. Lipid proteomics studies showed that phosphatidylcholine (PC), phosphatidylethanolamine (PE), and mitochondrial-specific phosphatidylcholine (CL) in MEF cells were significantly oxidized after GPX4 was knocked out, which led to mitochondrial outer membrane rupture and cell lipid peroxidation. Lipoxstatin-1 can significantly prevent lipid peroxidation induced by GPX4 knockout and inhibit cell ferroptosis in mice and humans [87].

Ferroptosis is essential in the pathogenesis of DOX-induced cardiomyopathy, and DOX can lead to mitochondrial iron overload and membrane lipid peroxidation in mice. Apoptosis, necrosis, apoptosis, and autophagy blockers exert no significant effect on mice. However, by preserving mitochondrial function, Fer-1 and the iron-producing agent DXZ significantly decreased DOX-induced cardiac injury and mortality. MitoTEMPO, a mitochondrial antioxidant, has been reported to diminish lipid peroxidation, decrease DOX-induced cardiomyopathy, and prevent ferroptosis [44]. By controlling the generation of ROS, mammalian targets of rapamycin (mTOR) can prevent the formation of lipid-derived ROS, protect cardiac cells from iron overload, and mitigate ferroptosis [54]. However, ferroptosis inhibitors are currently being treated systemically rather than specifically, which may have multiple side effects. For example, DFO, an iron chelating agent, is known to have a short half-life, need long-term subcutaneous infusions, and provoke ototoxicity and neurotoxicity. Deferasirox (DFX), an iron chelator, is associated with gastrointestinal and renal toxicity [88, 89]. Studies have shown that Chinese herbs and their active ingredients are effective in the treatment of tumors such as rectal cancer, gastric cancer, breast cancer, cardiovascular disease, kidney disease, and nerve injury diseases mediated by the regulation of the expression of GPX4, SLC7A11, and other proteins and ferroptosis sensitivity [90–92]. The mechanism of the occurrence and development of cardiovascular diseases is very complex, and Chinese herbs such as ginseng and ginkgo biloba have shown great potential in treating cardiovascular diseases and yielded good results. For example, resveratrol can inhibit ferroptosis by reducing oxidative stress levels and Fe^2+^ content, increasing GPX4 expression, and decreasing TfR1 expression in the myocardium of I/R rats and oxygen-glucose deprivation/reoxygenation (OGD/R) H9C2 cells [93].

Ruscogenin, a saponin derived from the root of the Chinese herb *Ophiopogon japonicus*, has a significant anti-inflammatory effect. It alleviates myocardial ischemia-induced ferroptosis by increasing the expression of BCAT1/BCAT2, activating the Keap1/Nrf2/HO-1 pathway, and upregulating GPX4 and downregulating ASCL4 expression [94, 95]. Britanin is an antioxidant and anti-inflammatory active ingredient purified from Inula japonica Thunb, which can mediate the AMPK/GSK3*β*/Nrf2 signaling pathway, increase the expression of GPX4, reduce ferroptosis, and improve myocardial I/R injury [96]. Moreover, astragaloside IV, an isolated substance from the water extract of Astragalus membranaceus, possesses anti-inflammatory, antioxidant, and immunity-boosting properties. It is conceivable for the Nrf2/SLC7A11/GPX4 signaling pathway to modulate ferroptosis [97, 98]. Ginsenoside, the active ingredient in traditional Chinese medicine ginseng, has anticancer, antioxidant, anti-inflammatory, immunomodulatory, hypoglycemic, and antidiabetic effects. Wang et al. found that ginsenoside GBE-5 could be involved in lipid metabolism, biosynthesis of unsaturated fatty acids, and regulation of ferroptosis by UHPLC–MS metabolomics [99]. In addition, salidroside is a phenolic compound that was obtained from the Chinese herb *Rhodiola* and was known to have antioxidant, antiaging, anti-inflammatory, free radical scavenging, enhanced abnormal lipid metabolism, reduced iron accumulation, inhibited ferroptosis, and heart and blood vessel protection properties [100–102]. Baicalin is a flavonoid component that was isolated from *Scutellaria baicalensis* Georgi and possesses anti-inflammatory, antioxidant, and antibacterial properties. Activation of the Nrf2 pathway could lessen hypoxia-induced myocardial cell damage as well as the expression of ASCL4 and the effects of ferroptosis which lead to decreased hypoxia-induced myocardial I/R damage [103–105]. Salvianolic acid B extract from *Salvia miltiorrhiza* Bunge has the effects of promoting blood circulation, removing blood stasis, and resisting inflammation and resisting aging. It is frequently used to prevent cardiac dysfunction diseases such as atherosclerosis, myocardial cell injury, myocardial hypertrophy, and myocardial infarction. By controlling the Nrf2 signaling pathway, reducing lipid peroxide accumulation and mitochondrial damage, regulating the expression of myocardial iron homeostasis proteins and oxidative stress reactions, and inhibiting ferroptosis, it can significantly decrease myocardial injury in rats with myocardial infarction [106–108]. Last but not least, geniposide, an active compound mostly obtained from *Gardenia jasminoides* Ellis, exhibits anti-inflammatory, antioxidant, antithrombotic, and ameliorative effects on the disturbance of glucose and lipid metabolism. It has been used to treat a variety of chronic inflammatory disorders, and by activating GPX4, it can reduce myocardial infarction by regulating the oxidative stress and ferroptosis driven on by iron overload [109–112].

It is well established that cardiovascular disease is the leading cause of mortality, morbidity, and disability globally. Ferroptosis is a regulated mechanism of iron-dependent cell death. Iron overload, lipid peroxidation, and the breakdown of mitochondrial structure and function are the key features of ferroptosis. An increasing body of evidence from recently published studies suggested that iron excess significantly impacts the onset and progression of several cardiovascular illnesses, and ferroptosis could be a viable therapeutic target. It is the premise of clinical treatment and application to study the mechanism of ferroptosis. However, the molecular mechanism of iron death has not been fully clarified. There are several routes that contribute to ferroptosis, including the Nrf2 pathway, high levels of extracellular glutamic acid, iron metabolic problems, GSH depletion, GPX4 inactivation, and lipid peroxidation. To demonstrate the link between iron mortality and cardiovascular disease, more in-depth study is required. Ferroptosis also interacts with other programmed cell deaths. The crucial ferroptosis proteins GPX4 and SLC7A11 have also been implicated in other death modes, which need to be separated by future research. Despite the promising treatment of ferroptosis, ferroptosis inhibitors may have multiple side effects. Additionally, the majority of the medications were only utilized in experimental settings, for which the precise dosage and side effects were unclear. Traditional Chinese medicine, with its rich bank of natural resources, also demonstrates the traits of numerous forms of collocation, broad effectiveness, and relatively few negative responses when treating illnesses. The targeted treatment of cardiovascular diseases with traditional Chinese medicine alone or in combination with other ferroptosis-regulating drugs should be investigated to reduce its toxic and side effects due to their significant potential in the future. However, its specific action targets in cardiovascular diseases and the mechanism of regulating ferroptosis still need to be thoroughly studied. In conclusion, cardiovascular diseases are intimately correlated with ferroptosis, a novel type of cell regulatory death. A new challenge for improving the management and prognosis of cardiovascular diseases is ferroptosis regulation.
